# Polypropylene Production Optimization in Fluidized Bed Catalytic Reactor (FBCR): Statistical Modeling and Pilot Scale Experimental Validation

**DOI:** 10.3390/ma7042440

**Published:** 2014-03-27

**Authors:** Mohammad Jakir Hossain Khan, Mohd Azlan Hussain, Iqbal Mohammed Mujtaba

**Affiliations:** 1 Department of Chemical Engineering, Faculty of Engineering, University of Malaya, 50603 Kuala Lumpur, Malaysia; E-Mail: jakirkhanbd@gmail.com; 2 UM Power Energy Dedicated Advanced Centre (UMPEDAC); Wisma R & D, University of Malaya, 59990 Kuala Lumpur, Malaysia; 3 Chemical Engineering Division, School of Engineering, University of Bradford, Bradford BD7 1DP, UK; E-Mail: I.M.Mujtaba@bradford.ac.uk

**Keywords:** polypropylene, process parameter, optimization, fluidized bed reactor

## Abstract

Propylene is one type of plastic that is widely used in our everyday life. This study focuses on the identification and justification of the optimum process parameters for polypropylene production in a novel pilot plant based fluidized bed reactor. This first-of-its-kind statistical modeling with experimental validation for the process parameters of polypropylene production was conducted by applying ANNOVA (Analysis of variance) method to Response Surface Methodology (RSM). Three important process variables *i.e.*, reaction temperature, system pressure and hydrogen percentage were considered as the important input factors for the polypropylene production in the analysis performed. In order to examine the effect of process parameters and their interactions, the ANOVA method was utilized among a range of other statistical diagnostic tools such as the correlation between actual and predicted values, the residuals and predicted response, outlier *t* plot, 3D response surface and contour analysis plots. The statistical analysis showed that the proposed quadratic model had a good fit with the experimental results. At optimum conditions with temperature of 75°C, system pressure of 25 bar and hydrogen percentage of 2%, the highest polypropylene production obtained is 5.82% per pass. Hence it is concluded that the developed experimental design and proposed model can be successfully employed with over a 95% confidence level for optimum polypropylene production in a fluidized bed catalytic reactor (FBCR).

## 1. Introduction

Polypropylene is a type of thermoplastic polymer resin and a superior quality polymer material that originates from olefins [1,2]. Polypropylene and its composites have been given priority over all other polymers by engineers due to its diversified applications [3] from household stuffs to a wide range of industrial appliances [4], as structural plastic or a fiber-type plastic. A number of conventional materials like steel, aluminum wood *etc.* have also been replaced by polypropylene and its composites since their superior physical and chemical properties such as their light weight, sophisticated structural stability, greater dielectric vitality, better mechanical strength, corrosion resistance capability and flexibility are superior to these traditional materials [5,6]. However, polypropylene and its composites hold only 20% share of the gross world polyolefin production [7] and hence an optimization study on polypropylene production is important from a scientific and economical point of view to enhance its usages and to improve its share of the market. For its production, fluidization is considered a well-established technology used in most cases. The capability to carry out a variety of chemical reactions, homogeneous particle mixing and extra ordinary mass and heat transfer characteristics are some of the major advantages of using Fluidized Bed Catalytic Reactors (FBCR) in industrial scale polypropylene production. Furthermore, the gas phase fluidization process has been recognized as an environmental friendly and convenient technology by a number of researchers [8–10]. Very important operating conditions like temperature, pressure and composition can influence significantly the process of polymer fluidization and these operating conditions are required to be controlled to produce different grades of polyolefin [11,12]. Being an exothermic reaction, propylene polymerization generates heat when the reaction starts, which principally influences the other operating factors and product quality. As a result of these mechanisms, proper process modeling to cater for these complicated reactions, hydrodynamic aspects as well as mass and heat transfer in the fluidized bed reactor, is necessary to engage engineers and scientists to design technically efficient and operationally feasible reactors for these facilities [13–15]. Furthermore, the optimization of these operating parameters also requires functional relationship among the process variables through available process modeling techniques.

A classical model for the chemical engineering process which comprises chemical kinetics, physical property interactions, mass and energy balances is made up of a number of differential as well as algebraic equations for both dynamic and steady state processes [16,17]. Some researchers considered the polyolefin reactor as a well-mixed reactor and only proposed a purely mathematical model where the temperature and monomer concentration in the reactor were calculated [18–20]. On the basis of a mixing cell framework a comprehensive mathematical model has also been proposed for simulation of the transient behavior of a fluidized bed polypropylene reactor by using a steady state population balance equation coupled with the proposed dynamic model along with incorporation of multisite polymerization kinetics of multi-monomer [21]. Ibrehem *et al.* [22] recently proposed that emulsion and solid phases are the stages where polymerization reactions take place during fluidization and report that alteration of catalyst particles with different porosity affects the rate of reaction and hence their model was obtained taking these effects into consideration. However, all these models generally take into account partial assumptions on reaction rates which do not cover all reaction conditions and circumstances and are normally not validated experimentally. Furthermore, it is also challenging to formulate precise mathematical models to take all these operation and design aspects into consideration for such a complex polymerization process [23].

Another feasible modeling approach is through statistical techniques that have been applied by a number of researchers with the purpose of predicting the optimum operating conditions in chemical processes to obtain the highest yield of desired product [24–26]. In fact, Response surface methodology (RSM) has been described as a very functional statistical tool for determination of optimum processes parameters for lab scale to industrial scale, as highlighted by variousworkers [27–29]. RSM covers experimental design, process optimization and empirical modeling where targeted response may fluctuate with numerous process variables (termed factors). RSM is principally appropriate for problems where the explanation of the process mechanism is inadequate and difficult to be characterized by first-principles mathematical models. Being contingent on definite objectives, in reality these RSM methods generally vary in the experimental design system, the selection of appropriate models and the mathematical equations of the optimization problem. Thus a precise design of experiment (DoE) is vital for a prolific experimental study [30]. Classical factorial and central composite designs can be utilized to investigate the interactions of process factors depending upon the polynomial models obtained in this method.

However, from literature studies, no work has been reported so far for the optimization of process variables of propylene polymerization in a fluidized bed catalytic reactor (FBCR) by applying these statistical modeling techniques. Also very few works have been reported on studying a pilot scale catalytic reactor although this is extremely important for predicting and validating the set of appropriate significant process variables and parameters for industrial use [18,22,31]. Hence, the objective of our work was to investigate the relationship among various operating parameters and to find out the optimum process parameters for propylene polymerization in a pilot scale fluidized bed using RSM modeling and Central Composite Design (CCD) technique. This novel pilot plant is a prototype of an industrial scale polypropylene production plant which is now in operation under management of the National Petroleum Corporation, Malaysia. Another novelty of our plant is that sampling of the gases in the system was conducted with an online Refinery Gas Analyzer (RGA). This type of real time and sophisticated sampling facility is globally very rare even in an industrial scale set up, although being highly necessary. To the best of our knowledge, this is the first attempt to conduct research on polypropylene production applying RSM for process parameter optimization under various parameter interactions in an original designed FBCR pilot plant.

## 2. Experimental Studies

### 2.1. Pilot Plant Description and Operation

**Figure 1.**
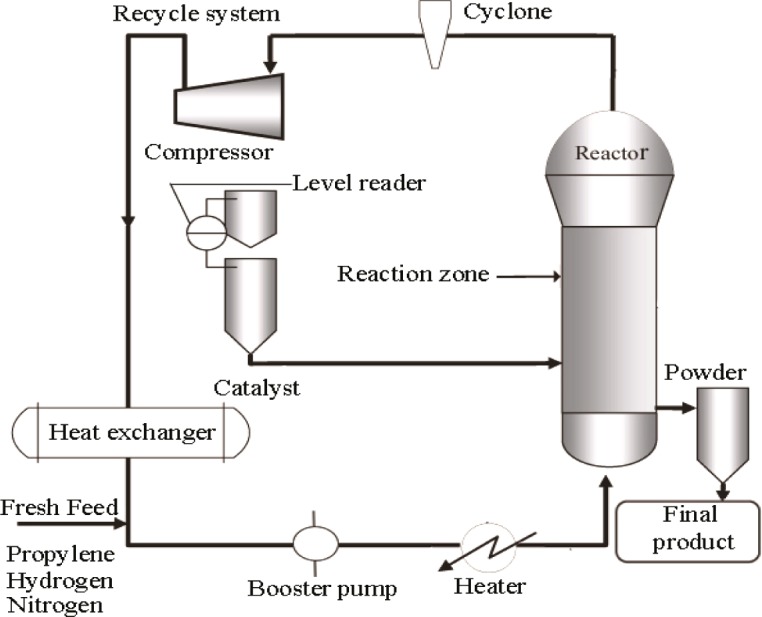
Schematic diagram of fluidization of the polypropylene production system.

**Figure 2.**
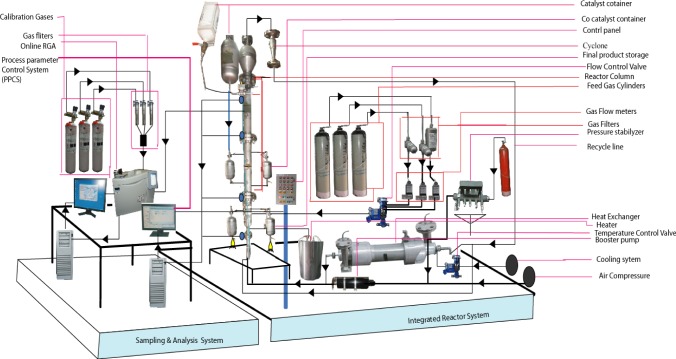
Detailed experimental set up of a pilot scale fluidized bed catalytic reactor (3D).

The pilot plant developed in our lab to produce polypropylene consists of a fluidized bed reactor zone and a disengagement zone designed for polymerization purposes, which is shown schematically in Figure 1 and its 3D figure shown in Figure 2. The inner diameter and height of the fluidized bed zones are 10 cm and 150 cm, respectively. The diameter is based upon the capacity of the production and the height of the reactor based on the fluid residence times. The disengagement zone has a diameter of 25 cm and a height of 25 cm. Catalyst particles were injected at 9 cm above the distributor plate located at the feed gas entrance point. In this polymerization reactor, the bubbling fluidized bed operates by the mixed gas fluidization process. Granulated polymer particle was used as the bed material because of its suitable mechanical stability. The operating temperature range in the center of the fluidized bed is maintained at about 70–80°C. A heater was used to regulate the gas inlet temperature of the reactor for startup condition to reach the required reaction temperature. Unreacted gas mixture from the top of the reactor is recycled and cooled by a shell and tube heat exchanger. One cyclone and four filters were fitted at the top of the reactor to remove fines entrained from the reactor. A buffer vessel was installed to control the pressure fluctuations in the system.

Propylene, hydrogen and nitrogen are used as the main input gases during the fluidization process which act as the medium of heat transfer as well as the reactants for the growing polymer particles during polypropylene production in the fluidized bed catalytic reactor. Continuous charging of catalyst and co-catalyst is carried out into the reactor which activates the reactants (propylene and hydrogen) to produce an outspread distribution of polymer particles. A co-catalyst is also used to keep the moisture below 2 ppm while activating the catalyst, which is the requirement for producing industrial grade polypropylene. After the bed has been fluidized, unreacted gases are separated in the disengaging section of the plant. The disengaged gases are recycled and mixed with fresh feed gases consisting of propylene, nitrogen and hydrogen This gas mixture passes through the heat exchanger in order to remove excess heat and is recycled through the gas distributor. The finished product is collected from the adjacent collection cylinder, whose connecting line is positioned just above the distributor plate. Propylene can be converted to polypropylene as much as 2%–3% per pass under fluidization conditions while the overall conversion can reach up to 98% [19,31]. The system is designed to run at a maximum pressure of 30 bar.

### 2.2. Pilot Plant Instrumentation

Temperatures in the reactor were measured at six different vertical positions, starting at 16 cm above the distributor plate. A temperature controller was used to control the temperature of the recycled gas entering the reactor. The air driven piston compressor was used to compensate for the pressure drop through the system. A flow meter and control valves were added just before the gas enters the reactor to regulate and measure the flow rate and circulation flow through the reactor system. The flow of catalyst was adjusted by a measuring valve, which revolves at a constant speed and inserts the catalyst into the reactor. Pressure and differential pressure indicators were placed at different points to check the pressure changes in the system and excess pressure is avoided by placing a relief valve on the top of the reactor set at 30 bar.

An online integrated Refinery Gas Analyzer (RGA) was used for analyzing the gas composition where wide-ranging automatic data recording devices and measuring equipment were employed in the pilot plant. The gas components consisting of hydrogen, nitrogen and propylene were analyzed online (with accuracy of ±0.03%) with a real time Refinery Gas Analyzer (RGA), a device of Perkin Elmer Clarus 580 series. The gas chromatography engineering software developed by Perkin Elmer was used for gas composition analysis which analyzes the multi component hydrocarbon and light gases. The three channel model in the data acquisition system provides a guaranteed analysis of the compositions of hydrogen, nitrogen, oxygen, carbon monoxide, carbon dioxide and propylene in approximately 8.5 min using two thermal conductivity detectors (TCD/TCD) and a flame ionization detector (FID).

### 2.3. Experimental Design and Optimization

In this study, the statistical analysis of propylene polymerization was performed using the Stat-Ease software where the CCD (Center Composite Design) was applied to analyze the interactions among the process variables and to identify the optimum process condition [32–34]. After collection of experimental data along with the design procedures, an empirical model was developed according to the RSM procedure. In this work, the polynomial function was fitted with the data at the initial stage after which the factor values were identified to optimize the objective function. The accuracy of the polynomial model fitting was determined by the coefficient of determination *R*^2^ and $R_{adj}^{2}$ in Equations (1) and (2) correspondingly:

$$R^{2}=1-\frac{SSQ_{residual}}{SSQ_{mod}+SSQ_{residual}}$$ (1)

Radj2=1−SSQresidualDgFresidual(SSQmod+SSQresidual)/(DgFmod+DgFresidual) (2)

The performance of the system was evaluated by analyzing the response of the percentage of propylene conversion per pass and the following is the mathematical equation related to the composite design, *i.e.*,

$$Y=\beta_{0}+\overset{k}{\underset{i=1}{\sum }}\beta_{i}\chi_{i}+\overset{k}{\underset{i=1}{\sum }}\beta_{ii}\chi_{i}^{2}+\overset{k-1}{\underset{i=1}{\sum }}\overset{k}{\underset{j=i+1}{\sum }}\beta_{ij}\chi_{i}\chi_{j}+\varepsilon$$ (3)

where, *Y* is the response vector, taking into account the main, pure-quadratic, and two-factor interaction effects while ε is the error vector. Regression and graphical analysis of the experimental design data and evaluation of the statistical significance of the various equations obtained were carried out in this analysis. The optimum preparation conditions were estimated through regression analysis and three-dimensional response surface plots of the independent variables with each dependent variable. Furthermore, the *p*-value is considered as a feature to measure the level of significance of all independent variables which at the same time signify the interaction intensity between all independent variables where the smaller *p*-value indicates the higher level of significance of the related variable.

The consequence of the second-order regression models was tested by the use of ANOVA and *F*-value analysis. This calculated *F*-value can be expressed from the following equation:

$$F=\frac{MnS_{RG}}{MnS_{RD}}$$ (4)

where the meaning of these terms can be referred to in the nomenclature section.

The *DgF* based F distribution for residual and regression is applied to compute the *F*-value in the particular point of importance. From these analyses, regression coefficients are obtained based on their significances with respect to the *p*-value.

The coefficient of variation (CV) indicates the extent of error of any model which is measured as the percentage of standard deviation over mean value given as:

$$\text{CV}=\frac{SD}{mean}\times 100$$ (5)

If the CV of a model does not exceed 10%, the model can be rationally regarded as reproducible.

## 3. Results and Discussion

### 3.1. Verification on Statistical Models

The independent variables considered important in this process are reaction temperature (A), system pressure (B) and hydrogen concentration (C). Reaction temperature refers to the temperature used during the initiation of the polymerization process, while system pressure refers to the required pressure of 20 bar process maintained at the starting point of reaction even though the system can be sustained at 30 bar. The range and coded level of the polymerization process variables studied are listed in Table 1. The independent variables were coded to the (−1, 1) interval where the low and high levels were coded as −1 and +1, respectively. According to the CCD, the total number of experiments required to be conducted is 20 runs. The polynomial equations were further used to plot three dimensional (3-D) surfaces and two-dimensional (2-D) contours to visualize the individual and interactive effects of the process factors on the response variables within their predefined ranges.

Batch experiments for 20 runs with different combinations of the process variables were carried out in the experiments. The percentage of polypropylene production was considered as the response. The proposed combination parameters for the experimental design and consequent results of the response using CCD are listed in Table 2. The Mean Square Error (*MnS_er_*) of the center point is 0.00005, which shows the accuracy of the data points taken and justifies the use of these data to obtain the model coefficients in Equation (6).

Experimental results showed that the polymer conversion ranged from 3.1%–5.82%. The maximum yield (5.82%) was found under the experimental conditions of A = 75°C, B = 25 bar and C = 2% which shows that for achieving the perfect coordination of experimental parameters for propylene conversion, the observation of precise optimum process conditions is mandatory.

### 3.2. Model Fitting

By the analysis of variance (ANOVA) method, the consequent *F*-value and *p*-value analysis were utilized. The summary of the Linear, Quadratic, 2FI (2 Factor Interaction) and Cubic model is shown in Table 3. The linear model represents the sequential sum of squares for the linear terms (A, B and C). The 2FI model implies the sequential sum of squares for the two-factor interaction terms (AB, BC and AC). The Quadratic model exhibits the sequential sum of squares for the quadratic (A^2^, B^2^ and C^2^.) terms. For all the above models small *p*-value (Prob > F) indicates that selected model terms can improve the model significance. The *F*-value is also associated with these models. The larger *F*-value indicates more of the variance can be explained by the model; a small number indicates the variance may be more due to noise.

It is observed from Table 3 that the quadratic model is the best fit model in terms of its significance and for this experimental design, the 2nd order model is suggested, as the *p*-value of this model is also smaller than that of other models.

For the proposed quadratic equation, the independent variables matched were also tested for the integrity of fit. The suitability of the fitted model was assessed using numerous indicators and the outcomes are presented in Table 4. To evaluate the appropriateness of the model, the R^2^, the $R_{adj}^{2}$ CV and *F*-value were used [35]. According to Table 4, the *F*-value of the model at 226.46 indicates the significance of this model, which also shows negligible tendency towards noise [36,37]. The probability value was found to be extremely low (*p*-value <0.0001) since less than 0.0500 for the *p*-value indicates that the model terms chosen are considerably important. The value for the coefficient of determination, *R*^2^ can be used to judge the precision and accuracy of the proposed model. The acquired value at 0.9951 specifies that 99.51% of the variability in the dependent variable could be justified through the model, and only 0.49% of the overall variations cannot be clarified [11,38]. Furthermore, the obtained value of the adjusted determination coefficient ( $R_{adj}^{2}$) is 0.9907, which shows a good relationship among the independent variables. In the current work, an incredibly low value of CV (1.75%) indicated a high level of accuracy and an excellent consistency of the model for the experimental results. The results shown in Table 4 prove that all the linear terms (A, B and C) and the quadratic terms (A^2^, B^2^ and C^2^) were important model terms due to their small *p*-value.

In order to show the significance of the individual parameters on the response, another effective statistical tool, *t*-test, was carried out. The *t*-test can show the level of significance of every individual parameter. From Table 5 it can be observed that the *p*-value obtained from the *t*-test analysis is much lower than 0.05 for every individual factor (A, B and C) which indicates that each of the factors (temperature, pressure and hydrogen [%]) taken into consideration is a highly significant factor for the polypropylene production process.

The subsequent second order polynomial equation was established by the application of least squares method and multiple regression study on the obtained data and given by Equation (6) below, *i.e.*,

$$\begin{array}{l} \text{Polymer conversion }(\%),\text{Y}=(0.13\times \text{A})+(0.12\times \text{B})-(1.05\times \text{C})+(0.044\times \text{A}\times \text{B})+\\ (0.024\times \text{A}\times \text{C})-(0.026\times \text{B}\times \text{C})-0.12\times {\text{A}}^{2}-0.40\times {\text{B}}^{2}-0.33\times {\text{C}}^{2}+5.19 \end{array}$$ (6)

where Y is the predicted percentage of polypropylene conversion, whilst A (temperature), B (pressure) and C (Hydrogen) are the coded form of independent variables of the model.

#### Diagnostic Statistics for Model Adequacy

**Figure 3.**
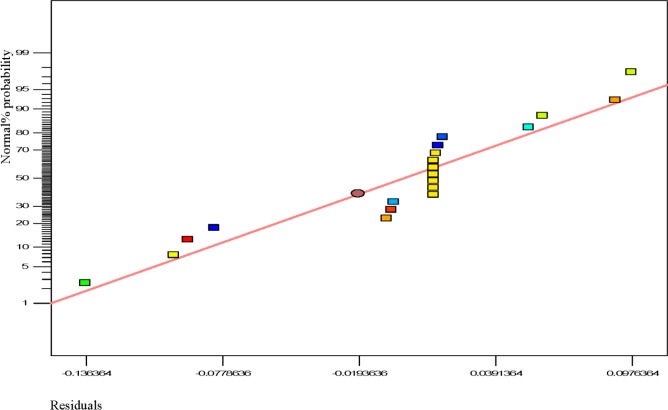
Normal probability plot.

**Figure 4.**
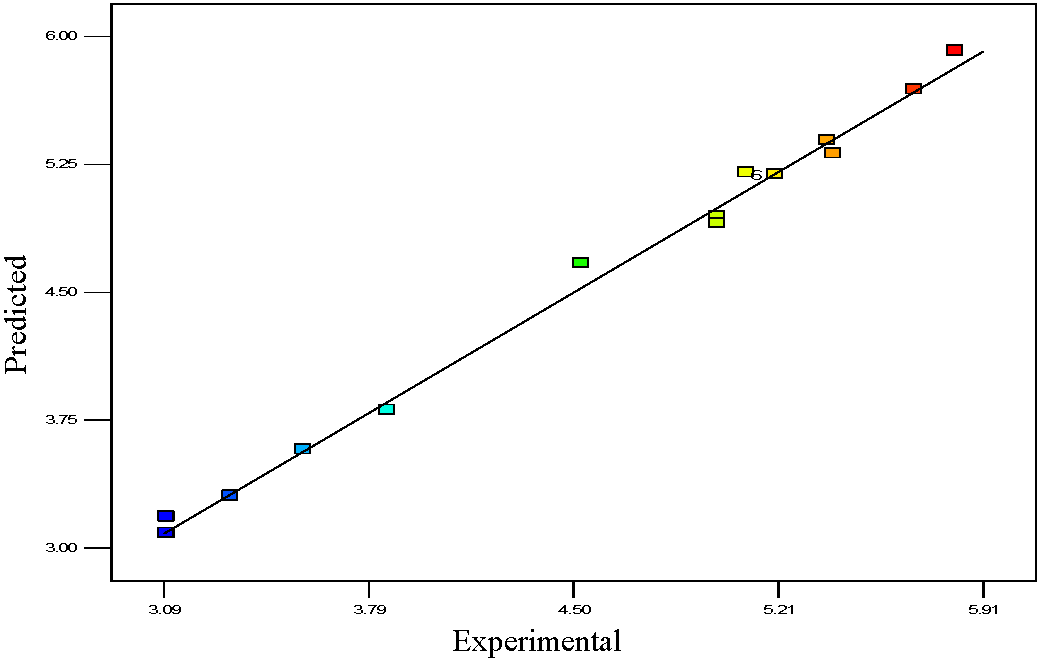
Linear correlation between actual and predicted values.

Usually, it is essential to confirm first whether the fitted model provides an adequate approximation of the actual values or not. Even though the model explains an acceptable fit, further continuation of the analysis and optimization of the integrated response surface tends to prevent inadequate or misleading results. In this study, several diagnostic tools were used to check the adequacy and the process parameters. The appropriateness of the models was also estimated by the influence plots and the residuals (difference between the anticipated response value and the actual value) in order to determine the coefficient for the data obtained experimentally in this work. Residuals are usually considered as components of variations, imprecisely fitted to the model and subsequently it is predicted that they behave according to a normal distribution feature. For the evaluation of normality of the residuals, a graphical visualization of the normal probability plot is considered as the proper method. In Figure 3, the scrutinized residuals are plotted against the predicted values, where, they lie rationally close on a straight line and exhibit no digression of the variance. In this way, the normal distribution of data can be confirmed. Furthermore, the regression model was used to calculate the predicted values of the polypropylene production (%) which were compared with the experimental results shown in Figure 4. As demonstrated in Figure 4, there is a suitable relationship between the experimental values and the predicted values which are distributed comparatively adjacent to the straight line. This phenomenon proves that the presented regression equation used for fitting the data was appropriate, and the CCD model in conjunction with the experimental design is efficiently functional for optimization of the polypropylene conversion (%).

**Figure 5.**
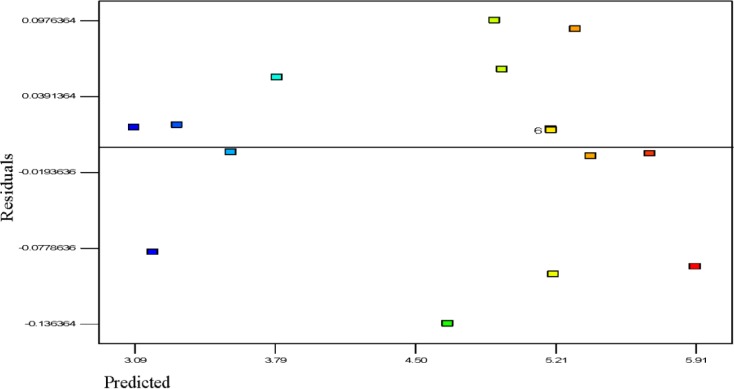
The residuals and predicted response plot for propylene polymerization.

Figure 5 shows the residuals and predicted polymerization capacity per pass of the batch reaction. The general trend is that the plot is scattered randomly, suggesting that the variance of the real findings is constant for every response value; the results indicate that the response variable does not require any modification since this result does not indicate any existence of large biased errors in the experiments performed. This can also be seen in the results of Table 2

**Figure 6.**
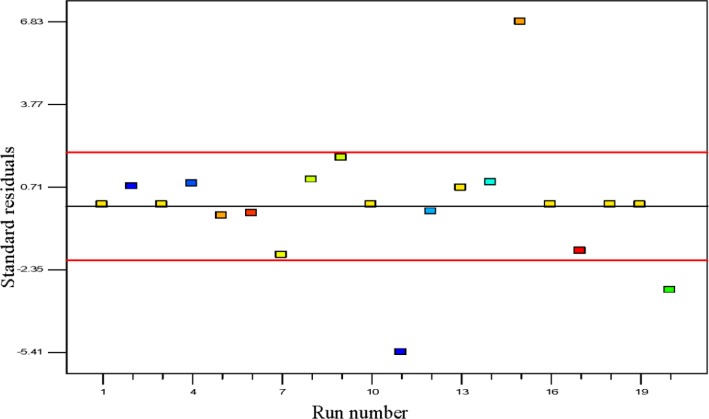
Outlier *t* plot for propylene polymerization per pass.

The outliers are cautiously tested in experimental design, since they may correspond to data acquisition error or rather more severe error [39]. The batch runs of polypropylene polymerization rate in percentage per pass are shown in the outlier *t* plot in Figure 6. The plot of outlier *t* is a calculation of the degree of the standard deviation, *i.e.*, intensity of deviation of actual value from the predicted value. Maximum standard residuals are required to be in the range of ±3.50 and any observed value alongside a standardized residual beyond this value is not totally related to its experimental response [40]. In this study almost all values for outlier *t* are lower than the interval of ±3.50 which proves that the estimation of the fitted model against the response surface is justifiably good enough without biased unknown errors. Only one data point was found to be beyond this value which contributed to the lesser significant term of the model [41].

**Figure 7.**
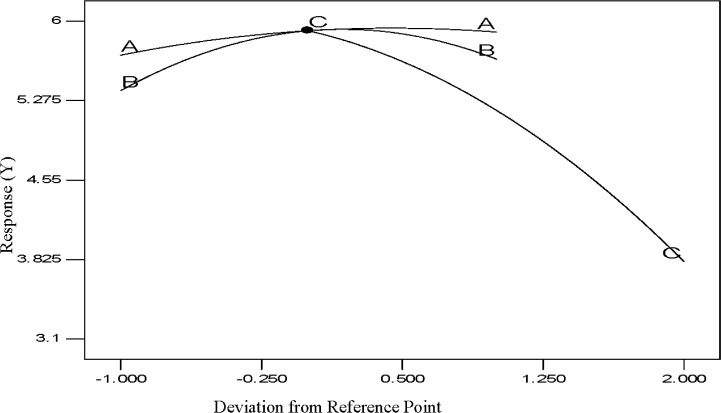
Deviation graph of process parameters.

The perturbation diagram for the polypropylene production rate with respect to the three input process factors is shown in Figure 7 where the influence of a process variable around a specific point in the design range is illustrated by this perturbation plot. In this method the response (the value of Y) is plotted with respect to only one variable of the overall process, one at a time over its range considering the additional process variables as remaining constant at their center point. A steep slope or curvature in a factor shows that the response is sensitive to that factor and a flat line demonstrates insensitivity to modification of that specific factor. The relative effects of every independent variable on the response (polypropylene production, %) can be seen in the perturbation plot of Figure 7. The sharp curvature of temperature (A), pressure (B) and hydrogen concentration (C) obtained, demonstrates that the propylene production (%) was responsive to all three process variables as expected. However, the perturbation analysis clearly shows that of the three parameters, hydrogen concentration (C) affects the value of Y more than the other two parameters as would be expected in such a process. This is also clearly shown for the value of coefficients as indicated in Equation (6).

### 3.3. 3D Response Surfaces and Their Corresponding Analysis

RSM provides several benefits for observing the effect of interaction within independent parameters and to recognize the effects of binary combination of linking two independent factors efficiently. However, it is easier to understand the interactions between factors graphically and the application of three-dimensional plots of the model is further useful for the graphical explanation of the interactions in this study [42]. Here the 3-D response surfaces were plotted by applying Equation (6) in order to show the polypropylene production rate which was affected by the various levels of other process variables. The interaction character between two process parameters can be explained by the response surfaces whilst the other process parameters remained constant at their center point. To identify the optimum levels of the process parameters, the 3D plot line can also be used to find the optimum response of polymer conversion yield at the highest point of the surfaces. In these figures, the color line levels indicate the various effects on the polypropylene production rate.

**Figure 8.**
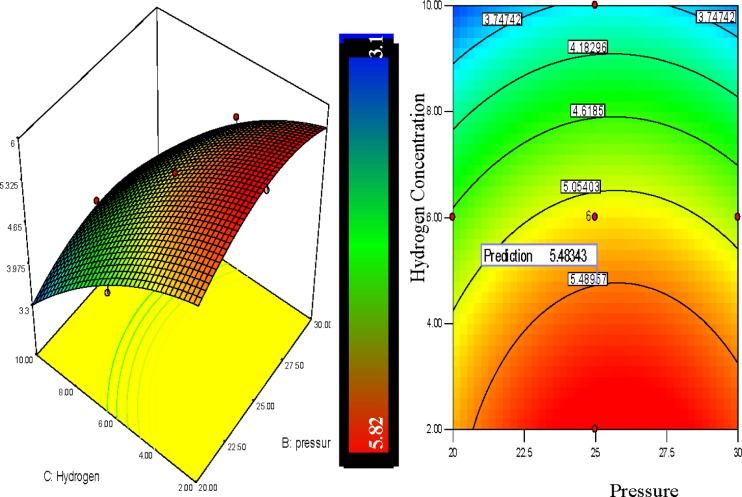
3D Response surface and contour plot of hydrogen concentration *vs*. pressure on polypropylene production (%).

The polypropylene production rises with the decrease of hydrogen percentage. It can be observed from Figure 8 that the hydrogen percentage showed a positive linear influence on the polypropylene production and the production increased notably in lower concentrated hydrogen regions. From the 3D graph of Figure 8 it is depicted that the combination of temperatures of 75°C, pressure of 25 bar and hydrogen of 10% shows a 3.86% polypropylene production per pass whereas at a temperature of 75°C and pressure of 25 bar with hydrogen of 6% and 2% the polypropylene production is shown at 5.2% and 5.82% respectively.

Figure 8 shows the response surfaces of the combined effect of hydrogen concentration and pressure on polymer conversion. Hydrogen concentration and system pressure both showed a positive effect on polypropylene production. From the contour plot, it can be clearly seen that decrease of hydrogen concentration increases the polypropylene production percentage while the increase of pressure also speeds up polypropylene production. The red colour zone indicates the optimum results while the other colors shows the lower values of the response.

Hydrogen is well recognized for its role as a chain transfer agent in industrial scale polypropylene production. The initial insertion of hydrogen decreases the molecular weight of polypropylene, which increases the diffusion rate of monomer on to the catalyst active site. It has also been reported that the nature of catalyst, monomer, and reaction conditions can also significantly affect the hydrogen effect on polypropylene production [43,44]. Researchers have also shown that the polypropylene polymerization rate significantly increases due to the increase in hydrogen concentration in the system up to a certain extent but a further increment of hydrogen concentration did not show any change in the polypropylene production rate [45]. The adsorption of hydrogen onto the catalyst surface was identified as the cause of this phenomenon.

In the literature, the local bed pressure variation has been reported as one of the major parameters for olefin polymerization in gas phase catalytic fluidization [18,31,46]. The reason is that pressure fluctuations can influence the effect of the dynamic phenomena taking place in the fluidized bed, such as from gas turbulence, bubbles hydrodynamics, and bed operating conditions [18]. The effect of pressure can also significantly affect the fluidized bed polymerization through the minimum fluidization velocity and particle size [6–19]. Naturally a pressure increase raises the inlet gas momentum and reduces the bubble surface tension, which boosts the disengagement of the bubble. The pressure intensification also enhances the fluid viscosity and reduces the buoyancy force, which slows down the detachment of the bubble from the particles [47].

## 4. Conclusions

The optimum experimental conditions for the production of polypropylene in a pilot scale fluidized bed catalytic reactor (FBCR) was verified by response surface methodology coupled with central composite design. The set of equations and predicted values from the statistical model were compared with experimental data. Independent variables, namely temperature, pressure and hydrogen concentration, were identified as the most important parameters that need to be determined to optimize the polypropylene production. The optimum condition for polypropylene production from this study was found to be at a temperature of 75°C, pressure of 25 bar, and hydrogen concentration of 2%. The projected polypropylene production from the statistical model was found to be at 5.2%, whereas from the experimental data gave 5.82%. Correlation between system pressure and reaction initiation temperature shows interaction between them and the outcome of various statistical techniques applied in this study proved that the proposed model is an excellent alternative to conventional first principle models. Finally we can conclude that the excellent correlation coefficients obtained for the developed correlations for the three responses can be successfully used with over 95% confidence, for operation of the process to produce optimum polypropylene production in the real plant. This would in turn accelerate the global usage and availability of this versatile plastic which is inexpensive and an excellent alternative for many other materials in the market.

## Figures and Tables

**Table 1.** Coded levels for independent variables used in the experimental design.

Factor	Name	Units	Type	Low Coded	High Coded	Low Actual	High Actual
A	Temperature	°C	Numeric	−1.000	1.000	70.00	80.00
B	Pressure	bar	Numeric	−1.000	1.000	20.00	30.00
C	Hydrogen	%	Numeric	−1.000	1.000	2.00	10.00

**Table 2.** Central Composite Design (CCD) experimental design and results of the response surface.

Run	Factor A, Temperature (°C)	Factor B, pressure (bar)	Factor C, Hydrogen (bar)	Response, Y, Polymer conversion (%) (Experimental result)
1	70	20	10	3.10
2	70	20	2	5.20
3	75	20	6	4.53
4	80	20	10	3.32
5	80	20	2	5.40
6	75	25	10	3.86
7	70	25	6	5.00
8	75	25	6	5.20
9	75	25	6	5.20
10	75	25	6	5.21
11	75	25	6	5.20
12	75	25	6	5.21
13	75	25	6	5.19
14	75	25	2	5.82
15	80	25	6	5.10
16	70	30	2	5.38
17	70	30	10	3.10
18	75	30	6	5.00
19	80	30	2	5.68
20	80	30	10	3.57

**Table 3.** Statistical parameters for sequential models.

Source	Sum of squares	Degrees of freedom	Mean square	*F*-value	*p*-value
Linear	11.39	3	3.80	21.55	<0.0001
2FI	0.025	3	8.446 × 10^−3^	0.039	0.9891
Quadratic	2.73	3	0.91	130.90	<0.0001
Cubic	0.066	4	0.016	28.79	0.0005

**Table 4.** Statistical parameters for sequential models.

Source	Sum of Squares	df	Mean Square	*F*-Value	*p*-value (Prob > F)
Model	14.14	9	1.57	226.46	<0.0001
A-Tempeature	0.17	1	0.17	23.98	0.0006
B-pressure	0.14	1	0.14	20.06	0.0012
C-Hydrogen	11.09	1	11.09	1597.78	<0.0001
AB	0.015	1	0.015	2.21	0.1683
AC	4.513 × 10^−3^	1	4.513 × 10^−3^	0.65	0.4388
BC	5.513 × 10^−3^	1	5.513 × 10^−3^	0.79	0.3937
A^2^	0.038	1	0.038	5.49	0.0411
B^2^	0.45	1	0.45	64.27	<0.0001
C^2^	0.30	1	0.30	42.56	<0.0001

Lack of Fit: 0.069; R-Squared: 0.9951; Adj. R-Squared: 0.9907; CV%: 1.75.

**Table 5.** *t*-Test result for testing the significance of individual parameters.

One-Sample Test (Individual Parameter)			
Factor	*t*	*DgF*	*p*-value
Factor, A	92.466	19	0.00001
Factor, B	30.822	19	0.00001
Factor, C	9.247	19	0.00001

## Notations

*SSQ*: sum of squares
*SSQ_mod_*: sum of squares of model
*SSQ_residual_*: sum of squares of residual
*p*: number of model parameters
*n*: number of experiments
ANOVA: analysis of variance
*MnS_RG_*: mean of square regression
*MnS_RD_*: mean of square residual
*MnS_er_*: Mean Square Error
*S_RG_*: sum of squares
*S_RD_*: sum of residual
*DgF_mod_*: degree of freedom of model
*DgF_residual_*: degree of freedom of residual
*R^2^*: determination coefficient
$R_{adj}^{2}$: adjusted coefficient of determination
CV: coefficients of variation
*F*-value: model significance
*SD*: Standard Deviation
